# Supplementary material for: BDNF in the Dentate Gyrus Is Required for Consolidation of “Pattern-Separated” Memories
Source: Cell Rep. 2013 Oct 24;5(3):759–68. doi: 10.1016/j.celrep.2013.09.027 (PMC3898274; doi:10.1016/j.celrep.2013.09.027)
Supplement: Document S1. Supplemental Analysis and Tables S1 and S2 [file mmc1.pdf]

Table S1. Total Exploration Times during the Sample Session for All Experiments

Fig n°	A1	A2	A3	A1	A2	A3
1C	36.2±3.5	38.5±1.7	44.2±3.7			
	IgG			anti-BDNF		
2C	28.3±4.0	33.0±5.0	33.9±6.8	27.7±5.1	33.7±3.9	27.7±5.1
	MSO			ASO		
3C	32.1±1.7	30.3±2.1	31.6±1.8	26.6±2.6	29.7±3.1	28.4±2.8
6B	26.7±2.2	26.8±2.6	25.7±2.4			

Results are expressed as mean ± SEM in seconds. A1, A2 and A3 represent the different locations as indicated in each one of the figures.

Table S2. Total Exploration Times during the Choice Session of the SLR Task for All Experiments

Results are expressed as mean ± SEM in seconds.

Fig n°	Novel location	Familiar location	Novel location	Familiar location	Novel location	Familiar location	Novel location	Familiar location
	Novel condition		Familiar condition					
1D	29.5±5.5	17.7±3.7	21.7±2.2	22.03±2.7				
	Small separation		Large separation					
1E	31.8±3.1	20.1±2.4	31.5±4.5	17.3±1.8				
	s-SLR				d-SLR			
	IgG		anti-BDNF		IgG		anti-BDNF	
2D	38.5±3.1	27.7±2.2	28.8±1.6	35.8±3.5	34.2±4.7	21.0±2.7	36.4±2.0	21.6±0.67
	s-SLR				d-SLR			
	IgG		anti-BDNF		IgG		anti-BDNF	
2E	26.1±2.2	17.4±1.5	23.0±2.3	25.5±3.5	37.4±4.6	21.3±2.8	37.4±2.4	22.7±1.3
2F	s-SLR							
	IgG		anti-BDNF					
	19.9±2.4	12.3±2.5	19.8±2.4	12.3±1.3				
	s-SLR				d-SLR			
	MSO		ASO		MSO		ASO	
3D	21.6±2.3	12.33±1.3	15.4±2.5	21.5±5.3	30.4±17.2	17.2±	31.2±5.7	18.2±3.3
	s-SLR							
	IgG		anti-BDNF					
4B	25.5±3.3	17.5±2.9	30.1±5.7	16.4±3.1				
	xs-SLR							
	Saline		BDNF					
6C	19.7±3.5	22.3±5.3	25.2±2.0	16.5±1.5				

Supplemental Analysis

In some experiments, namely those depicted in Figures 2D and 3D, infusion of the anti-BDNF antibody or the BDNF ASO in the s-SLR condition or saline in the xs-SLR condition produced numerically negative discrimination ratios. We thus analyzed whether these negative ratios were statistically significant. Both anti-BDNF and BDNF ASO values were not statistically different from zero ($p>0.05$ anti-BDNF vs 0 and $p>0.05$ BDNF ASO vs 0, One sample *t* test. In addition, we found that for the experiments shown in Figures 2D and 3D, control values were always significantly above zero ($p<0.01$ anti-IgG vs 0 and $p<0.001$ BDNF MSO vs 0, one sample *t* test). This analysis provides further evidence that the observed effects were due to the ability of the drugs to bring performance to chance levels, and were not driven by the apparent negative discrimination ratios observed in some instances.
