# Supplementary material for: BDNF in the Dentate Gyrus Is Required for Consolidation of “Pattern-Separated” Memories
Source: Cell Rep. 2013 Oct 24;5(3):759–68. doi: 10.1016/j.celrep.2013.09.027 (PMC3898274; doi:10.1016/j.celrep.2013.09.027)
Supplement: Document S2. Article plus Supplemental Information [file mmc2.pdf]

Cell Reports
Article

BDNF in the Dentate Gyrus Is Required for Consolidation of "Pattern-Separated" Memories

Pedro Bekinschtein,[1,3,*] Brianne A. Kent,[1] Charlotte A. Oomen,[1] Gregory D. Clemenson,[2] Fred H. Gage,[2] Lisa M. Saksida,[1] and Timothy J. Bussey[1]
[1]Department of Psychology and MRC and Wellcome Trust Behavioural and Clinical Neuroscience Institute, University of Cambridge, Cambridge 23EB, UK
[2]Laboratory of Genetics, Salk Institute for Biological Studies, La Jolla, CA 92037, USA
[3]Present address: Instituto de Biología Celular y Neurociencias, Facultad de Medicina, Universidad de Buenos Aires, Buenos Aires C1121ABG, Argentina
*Correspondence: pbekinschtein@fmed.uba.ar
http://dx.doi.org/10.1016/j.celrep.2013.09.027
This is an open-access article distributed under the terms of the Creative Commons Attribution License, which permits unrestricted use, distribution, and reproduction in any medium, provided the original author and source are credited.

SUMMARY

Successful memory involves not only remembering information over time, but also keeping memories distinct and less confusable. The computational process for making representations for similar input patterns more distinct from each other has been referred to as "pattern separation." In this work, we developed a set of behavioral conditions that allowed us to manipulate the load for pattern separation at different stages of memory. Thus, we provide experimental evidence that a brain-derived neurotrophic factor (BDNF)-dependent pattern separation process occurs during the encoding/storage/consolidation, but not the retrieval stage of memory processing. We also found that a spontaneous increase in BDNF in the dentate gyrus of the hippocampus is associated with exposure to landmarks delineating similar, but not dissimilar, spatial locations, suggesting that BDNF is expressed on an "as-needed" basis for pattern separation.

INTRODUCTION

For most people, memory is about time. It is easier to remember a set of items in a memory test if they are presented a few seconds before memory retrieval than if they are presented several hours before. When memory fails, as it normally does in old age or under pathological conditions such as Alzheimer's disease, this failure is reflected in the inability to remember over an extended period of time, although the ability to remember over a few seconds may remain intact. Increasingly, however, memory researchers are becoming interested in the ability not to remember over time but to keep memories distinct and resistant to confusion. If asked to remember where you parked your car this morning, yesterday morning, and the day before, the task is difficult not because you need to remember over a long period—you can easily remember many things that happened 3 days ago—but because the similar memories of your car in that same parking lot are so easily confused. The ability to separate the components of memories into distinct complex memory representations that are unique and less easily confused has been simulated by computational models of memory and a putative mechanism by which this occurs has been referred to as "pattern separation." These computational models and subsequent experimental work have suggested that this crucial memory function may be localized to the dentate gyrus (DG) of the hippocampus (Gilbert et al., 1998; Leutgeb et al., 2007; McHugh et al., 2007) and, in particular, to the adult-born immature neurons in this substructure (Aimone et al., 2009; Clelland et al., 2009; Nakashiba et al., 2012). However, information on molecular interactions with these neurons in the service of this process is not yet available. In this set of studies, we hypothesized that brain-derived neurotrophic factor (BDNF) might be part of an essential mechanism underlying the consolidation of pattern-separated memories (Bekinschtein et al., 2011).

To test these specific ideas, we modified an established paradigm, spontaneous location recognition (Ennaceur et al., 1997; Warburton et al., 2000), to allow parametric manipulation of the load on pattern separation. We are aware the term "pattern separation" refers, in the original computational literature, to a specific proposed mechanism involving the transformation of an input representation to an output representation, in which the output is less correlated than the input, resulting in nonoverlapping stimulus representations. Our behavioral tests assess the use of such representations. However, it should be emphasized that our tests do not assess the mechanism of pattern separation, as defined by the computational modelers, directly. As pattern separation is thought to happen during encoding/consolidation stages of memory formation, the similarity of the to-be-remembered locations was varied during the encoding/consolidation, rather than retrieval phase of the task. Unlike other tasks used to study pattern separation, the use of a continuous variable as a measure of performance yields sufficient data within a single trial to allow manipulations at different stages of memory. In contrast, previous tasks using discrete trial procedures require many trials to collect sufficient data, and thus such manipulations would have to be repeated an impracticable number of times.

Cell PRESS
Open ACCESS

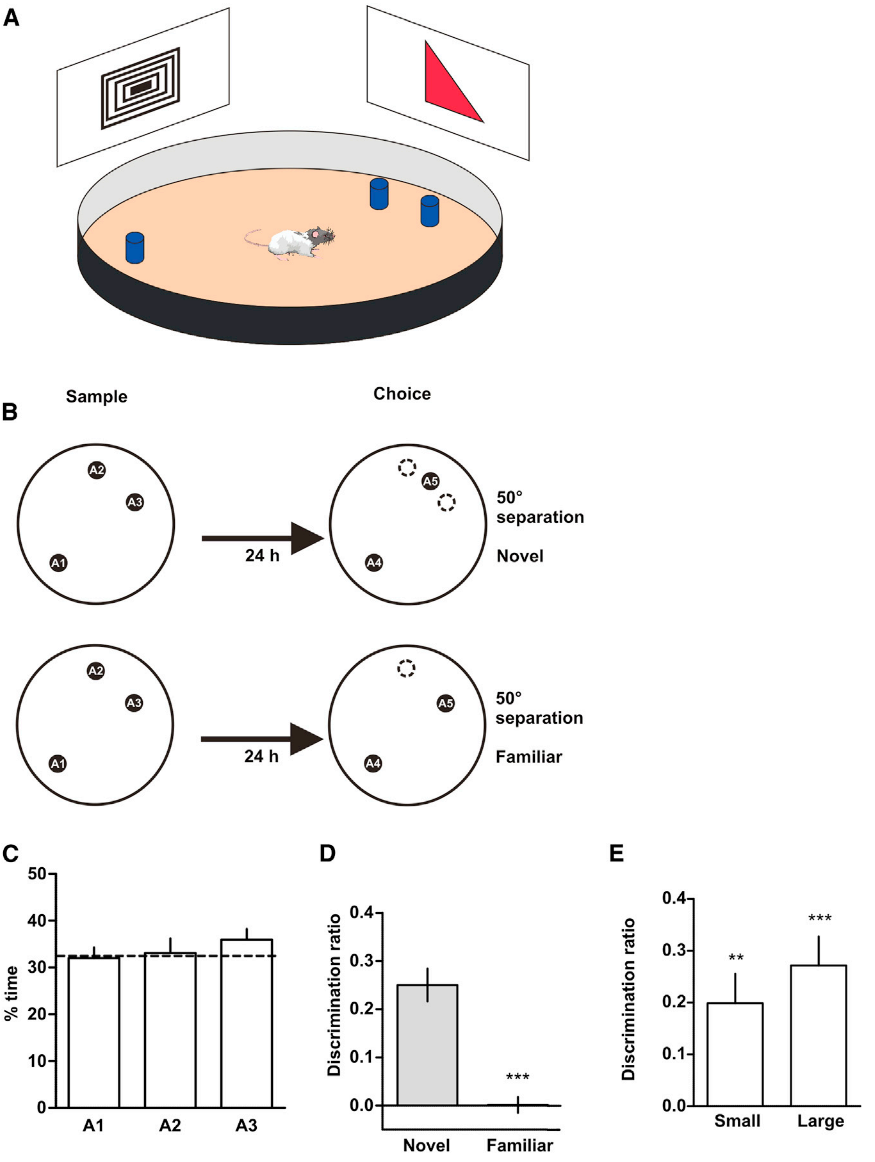

Figure 1. The Spontaneous Location Recognition Task

(A) Cartoon depicting the apparatus used for the SLR task. Two or three objects were used according to the different conditions in which the task was run. Walls are drawn shorter than actual size for illustrative purposes.

(B) Schematic of the SLR.

(C) Percentage of time exploring each of the locations during the sample phase of the SLR task. Rats spent equal amount of time exploring each of the three locations during the sample phase. This indicates that the differences in the discrimination ratio cannot be explained by preferential exploration of the more separated location (A1) during the sample phase.

(D) Discrimination ratios during the choice phase for the novel and familiar conditions. ***$p < 0.001$; $n = 8$.

(E) Discrimination ratios during the choice phase 24 hr after the sample phase for trials in which object A5 was kept in a familiar location whereas A4 was moved either a small (50°) or a large (120°) distance. Discrimination ratios were significantly different from zero. **$p < 0.01$, ***$p < 0.001$, one-sample t test; $n = 7$.

Data are expressed as the mean ± SEM.

Using our modified paradigm, we focused our initial enquiries into the molecular mechanisms on BDNF. BDNF is a small dimeric protein involved in both synaptic (Kang et al., 1997; Korte et al., 1995; Pang et al., 2004) and structural plasticity (Bamji et al., 2006; Tyler and Pozzo-Miller, 2001) in the adult brain, and it has been extensively shown that BDNF is required for memory processing (Bekinschtein et al., 2007; Cunha et al., 2010; Lee et al., 2004; Mizuno et al., 2000) and, in particular, consolidation (Lee et al., 2004). We thus hypothesized that BDNF might have an important role in pattern separation, in particular during the consolidation of pattern-separated memories.

RESULTS AND DISCUSSION

In the original spontaneous location recognition (SLR) task (Ennaceur et al., 1997; Warburton et al., 2000), rats are exposed during a sample phase to two identical objects placed in two different locations within an arena surrounded by distinct spatial cues. After a variable delay, rats are given a choice phase in which one of the objects was displaced to a novel location. Because rats naturally prefer novelty, they spend significantly more time exploring the novel location than the familiar one (Warburton et al., 2000). Our modified version of the task consisted of a sample (study) phase in which rats were exposed to three identical objects; two of them were close together and the third one was further away (Figure 1B). In this way, the similarity of locations could be manipulated at the time of encoding/consolidation, when pattern separation is thought to occur, rather than at retrieval, as in other tasks used to assess pattern separation (Clelland et al., 2009; Gilbert et al., 1998). During choice (test), the subject was exposed to two identical objects: one in a novel location between and equidistant from the two close ones explored during the sample phase, and the other one in its original location (Figure 1B). During a sample phase (left), animals were exposed to three identical objects (A1, A2, and A3) for 10 min. Two of them were close together (A2 and A3) and the third one was further away (A1). Twenty-four hours later, the rats were exposed during a choice phase (right) to two identical objects (A4 and A5), but one of the objects was displaced to either a novel location ("novel" condition) or remained in a familiar location ("familiar" condition). Dotted circles in Figure 1B indicate the location of

objects A2 and A3 during the sample phase. The familiar condition controls for the possibility that rats might explore the novel location more just because there is a change in the number of objects between the sample and the choice phase. The rats should not show a preference for either of the locations, since both of them were familiar. In different conditions in this task (see following experiments), the similarity of the two similar locations was varied (by varying the distance between the objects), but during the choice phase, when the animals' performance was being assessed, the testing situation was identical across the different conditions of the experiment. This is a better-controlled procedure than that used in previous methods in which the testing situation differs across conditions. The rationale behind the task was that if the rats "pattern separated" the two close locations, the representations of the two close locations should be distinct and resistant to confusion; therefore, the rats should show preference for the novel location. However, if the representations of the two locations were not sufficiently separated, presentation of the new and the repeated close locations would activate the same representation in memory and would thus not be distinguishable. The result would be that rats should behave as if the new location was familiar. Initial experiments found that normal rats, 24 hr after the sample phase, showed a significant preference for the object in the novel location, but not for the object that remained the same location as in the sample phase (Figure 1D). In a subsequent control procedure, we tested whether rats treated locations that were close together differently from objects that were far from each other, for example by using different cues to encode them. If this were the case, then displacement of the distant object would most likely result in different discrimination ratios than displacement of one of the close objects. However, we found that if one of the close objects (A5 in Figure 1, top panel) remained in a familiar location but the distant object (A4) was displaced instead either a small distance (50°) or a large distance (120°), then 24 hr later the rats preferred to explore the novel location and the discrimination ratios were similar to the ones obtained with the standard version of our task (Figure 1E; see Figures 2 and 3). This finding provides evidence that rats encode the close locations and the distant locations using similar cues and strategies.

BDNF has been shown to be essential for memory consolidation in several kinds of tasks (Alonso et al., 2002; Lee et al., 2004; Linnarsson et al., 1997; Mizuno et al., 2000), and thus we focused our experiments first on the consolidation as well as the encoding phase of the task by blocking BDNF function in the DG with a BDNF-blocking antibody either before or after the sample phase. This strategy has been successfully used previously to inhibit BDNF action in different behavioral paradigms (Bekinschtein et al., 2007; Peters et al., 2010). We used our task to test the hypothesis that blocking BDNF function in the DG during the sample phase (during encoding) should impair future retention of the SLR task only in the case where two of the locations are similar (spatial representations need to be pattern separated), but not if the locations are dissimilar (spatial representations do not need to be separated). To test this hypothesis, rats were injected with a BDNF-blocking antibody into the DG before or after the sample phase. For this experiment, the SLR task was run in two different ways by manipulating the separation between the locations to create two conditions with differing loads on pattern separation (Figure 2A). In the "similar SLR" (s-SLR) condition, two of the locations were separated by a 50° angle and the third one by a 155° angle from the other two (small separation; Figure 2A), and in the "dissimilar SLR" (d-SLR) condition, the three locations were separated by a 120° angle (large separation; Figure 2A). We reasoned that if the rats needed to pattern separate the two close locations in the s-SLR condition, but not in the d-SLR condition, then blocking BDNF DG should be impair performance only in the s-SLR condition. Infusion of a BDNF-blocking antibody into the DG 15 min before the sample phase impaired retention in the s-SLR condition 24 hr later compared to infusion of a control antibody (Figure 2D, left). In contrast, this treatment had no effect on the d-SLR version of the task (Figure 2D, right), indicating that BDNF was necessary for successful encoding, consolidation, or both only when the encoded spatial representations were similar, but not when they were different. To analyze whether BDNF was required for consolidation in the s-SLR condition, we infused BDNF-blocking antibodies into the DG 5 min after the sample phase (i.e., after encoding had already taken place). Inhibiting BDNF activity 5 min postsample impaired consolidation of the s-SLR task when assessed 24 hr later (Figure 2E, left). Again, we found no differences in retention scores between BDNF antibody and control antibody-injected rats when they were exposed to the d-SLR configuration (Figure 2E, right). The cellular consolidation process is a time-restricted process, with amnestic agents being effective only during a restricted time window (McGaugh, 2000). To test whether BDNF requirement for the s-SLR task was limited to the first few hours after the sample phase, BDNF-blocking antibodies were injected into the DG 6 hr after the sample phase. This treatment failed to impair retention of the s-SLR task 24 hr after the sample phase (Figure 2F), indicating that BDNF activity in the DG is required during a time-limited phase for memory consolidation of similar spatial representations. This result also rules out the possibility that the impairment found with pre- or postsample injections was due to a lingering effect of the drug during retrieval 24 hr later.

As with every spontaneous behavioral task, there might be a concern regarding a change in motivation to explore after a particular pharmacological manipulation; i.e., manipulations could change the animals' preference for novel items to familiar items. In our experiments, this factor could not account for the differences in the discrimination ratios, because that would mean that our manipulations of the DG somehow affected motivation only in the s-SLR condition, but not in the d-SLR condition. Moreover, the fact that infusion of the BDNF-blocking antibody 6 hr after the sample phase did not affect novelty preference in the s-SLR condition effectively rules out the possibility that a change in motivation explains these results. It is also unlikely that different strategies are used by the animal in the different conditions of the task. For example, the idea that the close objects might bias the rat toward using distal versus proximal cues seems unlikely, because a vast amount of data indicate that hippocampal dysfunction produces the opposite effect, namely impairment in the use of distal (allocentric), but not proximal (egocentric), cues (Burgess, 2008).

Cell PRESS
Open ACCESS

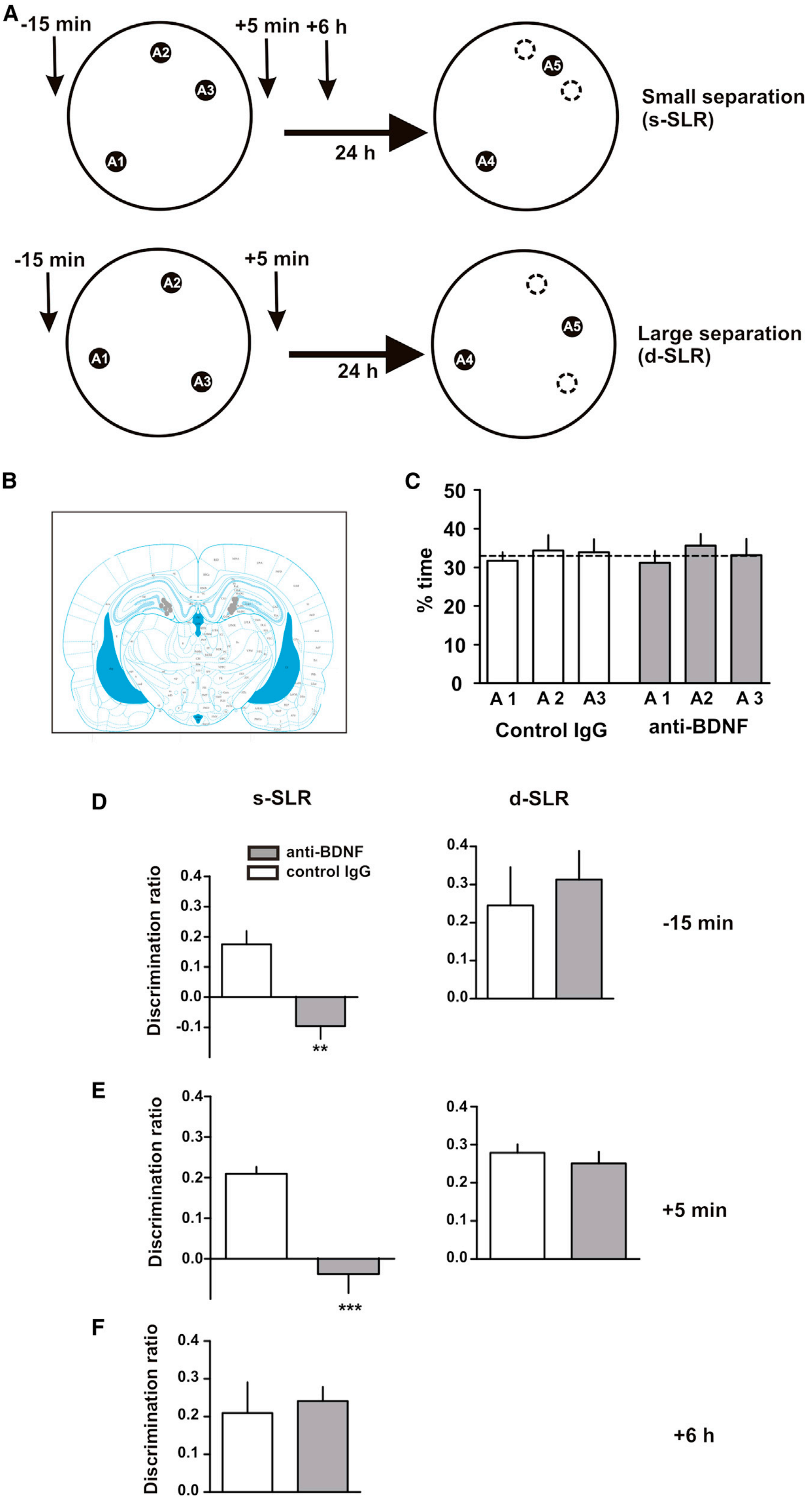

Figure 2. BDNF Activity in the DG Is Required for Memory Consolidation of Similar, but Not Dissimilar, Spatial Representations

(A) Schematic of the SLR task for the similar (s-SLR) or dissimilar (d-SLR) conditions depicting the time points at which IgG or anti-BDNF was infused.

(B) Coronal section indicating representative infusion sites in the DG.

(C) Percentage of time exploring each of the locations during the sample phase of the s-SLR task for control IgG- or anti-BDNF injected rats.

(D–F) BDNF antibodies or control IgGs (1 $\mu g\ \mu l^{-1}$ /0.5 μl side) were injected into the DG either 15 min before (D) or 5 min after (E) the sample phase. Injection of anti-BDNF into the DG 6 hr after the sample phase had no effect on s-SLR performance (F). $^{**}p < 0.01$, $^{***}p < 0.001$; n = 7.

Data are expressed as the mean ± SEM.

Cell PRESS Open ACCESS

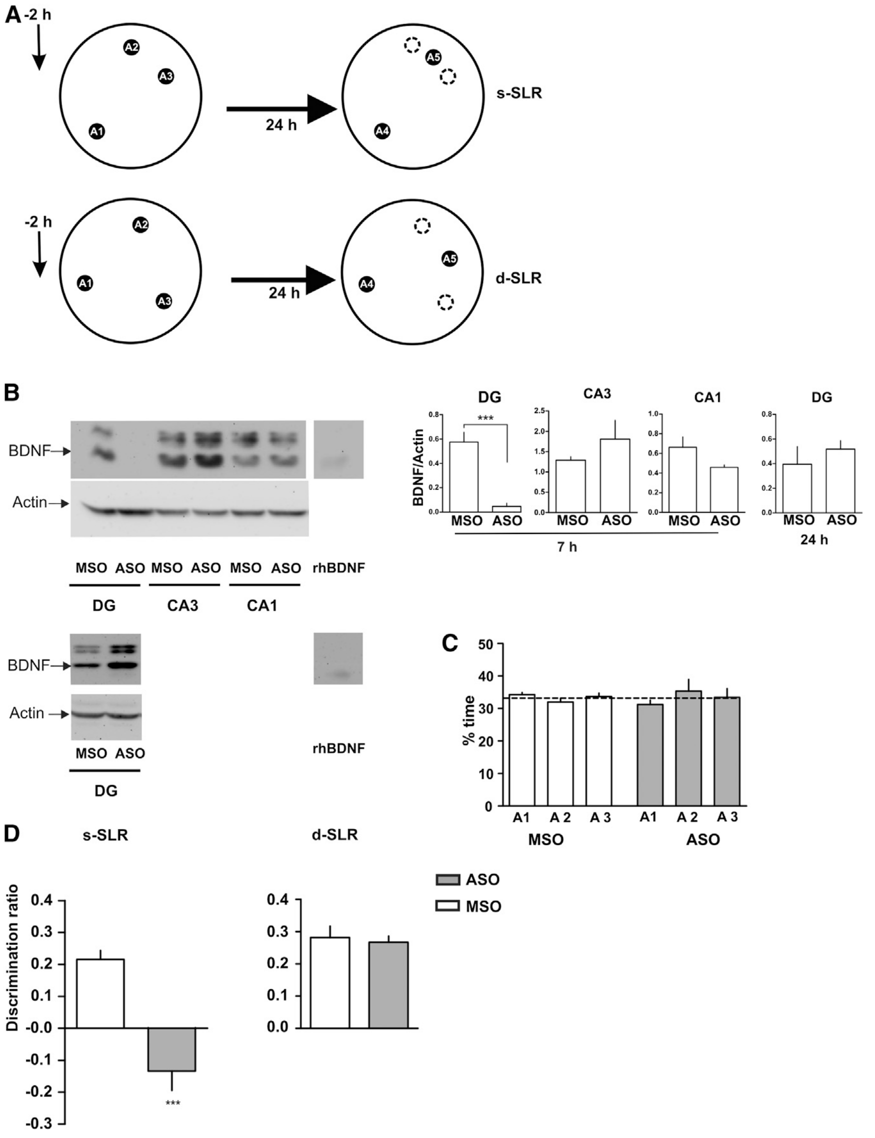

Figure 3. BDNF Expression in the DG Is Required for Memory Consolidation of Similar, but Not Dissimilar, Spatial Representations

(A) Schematic of the SLR task.

(B) Effect of the infusion of BDNF antisense oligonucleotides (4 nmol μl^{-1}/0.5 μl side; BDNF ASO) or BDNF scrambled missense oligonucleotides (4 nmol μl^{-1}/0.5 μl side; BDNF MSO) in the DG on BDNF steady-state levels 7 hr or 24 hr after injection. Top left: representative blots of BDNF and actin protein levels in the DG, CA3, or CA1 regions 7 hr after oligonucleotide injections. Bottom left: representative blots for BDNF and actin protein levels in the DG 24 hr after oligonucleotide injections. Right: quantification of BDNF expression after ASO or MSO injection. ***$p < 0.001$; n = 4.

(C) Exploration time during the sample phase or time spent exploring each of the locations 2 hr after BDNF ASO or BDNF MSO injection into the DG.

(D) Effect of the injection of BDNF ASO or BDNF MSO into the DG 2 hr before the sample phase during a choice phase 24 hr later in the s-SLR or the d-SLR version of the task. ***$p < 0.001$; n = 7. Data are expressed as the mean ± SEM.

To attempt to replicate this finding in a way that better ensures regional specificity, we used a different strategy to suppress BDNF function. Antisense oligonucleotides (ASO) can be designed to specifically target BDNF mRNA by preventing its translation, and this methodology has been proven to be very effective to specifically inhibit BDNF in vivo (Bekinschtein et al., 2007; Lee et al., 2004). Seven hours after infusion of 2 nmol of an antisense against BDNF (BDNF ASO) into the DG, basal BDNF levels were almost undetectable compared to infusion of a control scrambled missense oligonucleotide (BDNF MSO) (Figure 3B). In contrast, BDNF protein levels remained unchanged in CA1 or CA3 regions of the hippocampus (Figure 3B), even 7 hr after injection when the ASO would have time to spread considerably, indicating that BDNF knockdown was restricted to the DG. Twenty-four hours after infusion of BDNF ASO, BDNF expression in the DG was back to control levels (Figure 3B), indicating that the inhibition was transient and that the rats had normal levels of BDNF in the DG during the choice phase. Relative exploration of the three different locations during the s-SLR sample phase was not modified by injection of the BDNF ASO or BDNF MSO into the DG 2 hr before the sample (Figures 3A and 3C). However, BDNF ASO-injected rats showed no preference for the novel location during the choice phase 24 hr later compared to BDNF MSO-injected rats (Figure 3D). Negative discrimination ratios were not significantly different from zero (see supplemental analysis). In contrast, BDNF ASO injection had no effect on retention of the d-SLR task (Figure 3D). Again, the impairment in the s-SLR task cannot be explained by a protracted effect of BDNF ASO on retrieval during the choice phase, because 24 hr after injection of the BDNF ASO, BDNF in the DG was back to control levels.

Although we showed that BDNF in the DG was required for successful encoding/consolidation of similar, but not dissimilar spatial representations, it is also possible that BDNF is required during retrieval, that is, when a novel representation being encoded is compared to a similar one already stored in memory. To test this possibility, we infused BDNF-blocking antibodies into the DG 15 min before the choice phase in the s-SLR condition. We found no differences in discrimination ratios between anti-BDNF-injected rats and controls (Figure 4). This result suggests that, consistent with computational modeling (O'Reilly and McClelland, 1994; Rolls and Kesner, 2006) and the role of BDNF in memory consolidation (Lee et al., 2004), BDNF-dependent processing of pattern-separated memories occurs during the

Cell PRESS Open ACCESS

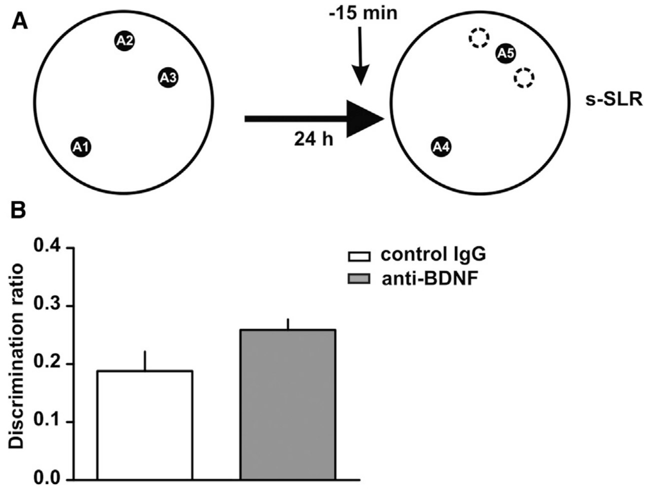

Figure 4. BDNF in the DG Is Not Required During Retrieval

(A) Schematic of the SLR task.

(B) Effect of BDNF antibodies (1 µg μl^{-1} /0.5 µl side) injected into the DG 15 min before the choice phase on the s-SLR task compared to control IgGs (1 µg μl^{-1} /0.5 µl side). $p > 0.1$; $n = 7$.

Data are expressed as the mean ± SEM.

encoding/consolidation phase, but not during the retrieval phase of memory processing.

The results of these experiments provide compelling evidence that BDNF in the DG is importantly involved in the molecular mechanisms underlying pattern separation. Moreover, they isolate the action of BDNF to the consolidation and perhaps also the encoding phase of memory, specifically. Particularly interesting is the finding that postsample injections, made after initial encoding of the to-be-remembered location, disrupt memory only in the s-SLR, but not in the d-SLR condition. This finding raises the question of whether BDNF is expressed equally in both conditions but only needed in the first, or whether BDNF is expressed on an ''as-needed'' basis, that is, spontaneously in response to encountering similar events, the representations of which need to be separated before storage in memory. To test this possibility, we exposed rats to two objects delineating either similar or dissimilar spatial locations within the open field. One group of rats was exposed to two identical objects separated by a 50° angle (small separation condition). A second group of rats was exposed to two identical objects separated by a 120° angle (large separation condition), and a control group was exposed to the empty arena (Figure 5A). One hour after the exposure, rats were sacrificed and the DG and CA1 regions were microdissected and homogenized for western blot analysis (Figure 5C). Immunostaining revealed a 3-fold increase in BDNF in the DG in the small separation group, but not in the large separation group (Figure 5D). No changes in BDNF protein content in CA1 were detected in either the small or the large separation group (Figure 5D). We found no differences between the two groups in total exploration time or time spent exploring each of the two locations (Figure 5B). There were no differences in the DG or CA1 in the protein levels of Zif268 (Figure 5D), another activity-regulated gene thought to be involved in the reconsolidation process (Lee et al., 2004), indicating that the changes in BDNF might be related to a consolidation-like process per se (Lee et al., 2004). These findings provide evidence that BDNF is expressed on an ''as-needed'' basis, that is, increased spontaneously in order to separate the representations of similar events. It is probably worth noting that in this experiment we used two objects instead of three—unlike in the memory experiments involving both sample and choice phases—to facilitate measurement of changes in BDNF specifically related to the separation between locations, avoiding potential confounds related to a third location. However, we did run a similar experiment using three objects and again observed a significant increase in BDNF in the DG 1 hr after exposure to the s-SLR configuration, but not in the d-SLR configuration. No significant changes were observed in the CA1 region (Figure 5E). This result replicates our previous finding and confirms that BDNF expression in the DG increases after exposure to the small separation under exactly the same conditions in which we performed the memory experiments.

Because BDNF has been shown to enhance memory when injected exogenously (Alonso et al., 2002; Peters et al., 2010), we tested whether exogenous human recombinant BDNF (rhBDNF) could enhance consolidation of similar representations. To be able to see memory enhancement, we brought control animals to chance performance by making discrimination more difficult. Two of the objects were brought closer together (40° separation) during the sample phase during which the animals explored the three locations equally (Figure 6A). Bringing the objects closer together did not allow control rats to recognize the new location as novel during the choice phase 24 hr later (Figure 6B). However, intra-DG injection of rhBDNF 5 min after the sample phase enhanced performance significantly with respect to saline-injected controls (Figure 6C), which suggests that BDNF is essential to begin the consolidation process of similar representations in the DG. Incidentally, these results also provide evidence that the animals do not use the objects themselves as spatial cues, because if they were using such proximal cues to guide their behavior, then the extrasimilar SLR (xs-SLR) should be easier than the s-SLR. However, vehicle-injected rats were not able to perform the task under these conditions.

In this study, we have shown that BDNF plays a role in pattern separation in the DG. What precisely might that role be? One idea is that for similar representations to be stored separately, the unique set of DG neurons that encoded the input patterns may need to stabilize and strengthen their connections with their outputs in CA3. During consolidation, these patterns of activity might be replayed and lead to activation of a program of gene expression that will eventually make these connections more stable (Karlsson and Frank, 2009). In this way, the neuronal ensembles that originally encoded the representations may be preferentially reactivated during retrieval 24 hr later. In this scenario, BDNF may promote plasticity in the activated encoded networks to strengthen the connections that will be reactivated during retrieval.

This study does not identify the population of DG neurons that BDNF acts upon, but combined with the finding that knockdown of neurogenesis impairs tasks thought to depend on pattern

Cell PRESS
Open ACCESS

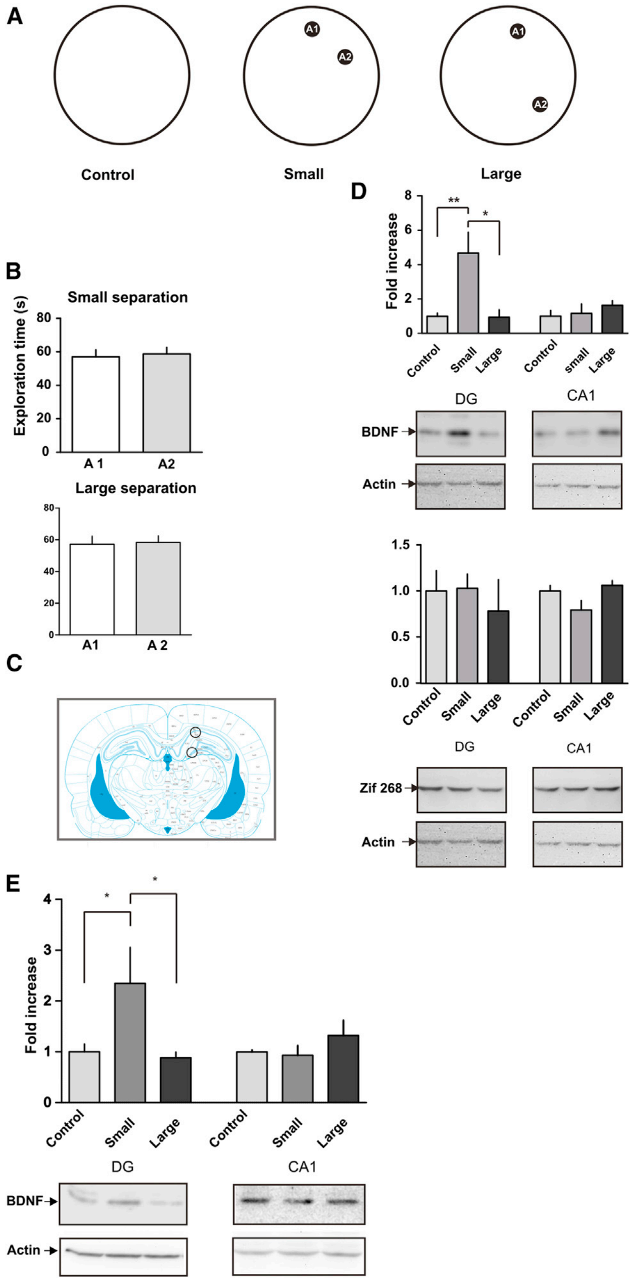

Figure 5. Exploration of Similar, but Not Dissimilar, Spatial Locations Is Associated with Increased BDNF Levels in the DG

(A) Schematic illustration of the task configurations.

(B) Total exploration time for each object in the small (top) and large (bottom) separation conditions.

(C) Coronal brain section at coordinate −3.96 from bregma depicting the areas isolated for BDNF protein measurements. Tissue was punched and homogenized for SDS-PAGE.

(D) Top: BDNF and actin protein levels in the DG and CA1 regions of rats subjected to the different conditions and corresponding representative blots. Bottom: Zif268 and actin protein levels in the DG and CA1 regions of rats exposed to the different conditions and corresponding representative blots. *$p < 0.05$, **$p < 0.01$; $n = 6$.

(E) BDNF expression in the DG and CA1 after exposure to three objects. We used the same conditions as in the sample phase during the SLR task. BDNF and actin protein levels in the DG and CA1 regions of rats subjected to the different conditions and corresponding representative blots. *$p < 0.05$; $n = 8$. Data are expressed as the mean ± SEM.

separation (e.g., Clelland et al., 2009), one possibility is that BDNF acts on adult-born immature neurons in the DG. By what mechanism that might occur is unclear. Although BDNF has been shown to increase survival of newborn neurons and increase neurogenesis (Rossi et al., 2006; Scharfman et al., 2005), it is unlikely that these processes underlie the effects seen in the present experiments. This is because the timings of the BDNF requirement for the task (minutes to hours) and development and incorporation of newborn cells into the circuits (weeks) are very different. Instead, the effect of BDNF is an acute one. Immature adult-born neurons have been shown to be more excitable than mature neurons and also to have enhanced plasticity (Ge et al., 2007; Schmidt-Hieber et al., 2004), and so they may respond more rapidly to inputs of ambiguous spatial information in the DG. This enhanced response may be very sensitive to BDNF levels present in the hippocampus. Indeed, it has been shown that ablation of tropomyosin receptor kinase B (TrkB) in progenitor cells has a significant effect on behavior and synaptic plasticity (Bergami et al., 2008). These results suggest that BDNF might be activating the TrkB receptor in immature neurons during pattern separation and that expression of BDNF might be the necessary stimulus for memory consolidation of similar representations to occur within the DG.

In summary, the present study has begun the investigation into the molecular events underlying the important mnemonic process of pattern separation. Starting with a focus on a candidate molecule, BDNF, we have shown that BDNF is critical for pattern separation but is not necessary for the identical task when the requirement for pattern separation is not high. We have demonstrated this by inhibiting BDNF action in the DG using two mechanistically distinct methodologies that impaired the discrimination of spatial representations only when the load for separation of representations was high. Furthermore, by using a behavioral paradigm that allows us to manipulate memory processing at different time points, we provide experimental evidence that (BDNF-dependent) pattern separation occurs during the encoding/storage/consolidation stage of memory processing. It does not occur during retrieval. In addition, post-sample injections of recombinant BDNF into the DG were able to enhance the separation of representations. Finally, our results suggest that BNDF is expressed in a spontaneous, as-needed manner when similar items that require separation are encountered. This is a surprising result, and the origin of the signal that determines this spontaneous release is an important target for future enquiry. Finally, the methods we have introduced to generate these findings may serve as a particularly useful tool for researchers interested in this important, emerging area of memory research.

EXPERIMENTAL PROCEDURES

Subjects

The subjects were 121 Lister Hooded rats (Harlan Olac) weighing approximately 250–300 g at the start of testing. The rats were housed on a reversed

Cell PRESS
Open ACCESS

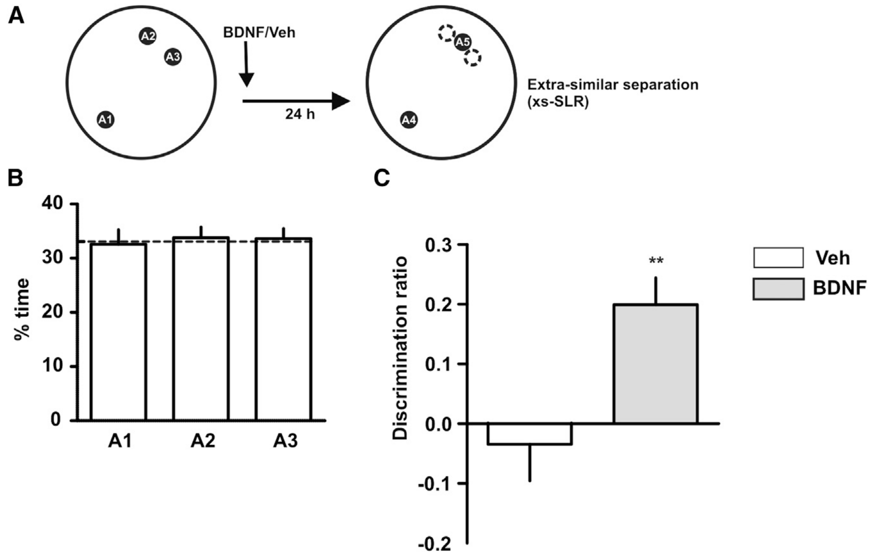

Figure 6. BDNF Enhances Consolidation of Similar Spatial Representations

(A) Schematic of the extrasimilar SLR task (xs-SLR). The task was similar to the s-SLR except that in the xs-SLR task, two of the objects were brought even closer together during the sample phase, resulting in poor performance of control animals during the choice phase 24 hr later.

(B) Percentage of time exploring each of the locations during the sample phase of the xs-SLR task.

(C) Effect of recombinant human BDNF (0.5 μg μl^{-1} /0.5 μl side; rhBDNF) or saline injected into the DG 5 min after the sample phase on performance during the choice phase 24 hr later. **$p < 0.01$; n = 7.

Data are expressed as the mean ± SEM.

12 hr light/12 hr dark cycle (lights on 19:00–07:00), in groups of two or four. All behavioral testing was conducted during the dark phase of the cycle. Rats were food deprived to 85%–90% of their free feeding weight, except during recovery from surgery, where food was available ad libitum. Water remained available ad libitum throughout. All experimentation was conducted in accordance with the UK Animals (Scientific Procedures) Act 1986.

Surgery and Cannulation

All rats were implanted bilaterally in DG of the dorsal hippocampus with 22G indwelling guide cannulas. Subjects were anaesthetized with ketamine (Ketalar, 90 mg kg^{-1}, intraperitoneally [i.p.]) and xylazine (Rompun, 6.7 mg kg^{-1}, i.p.) and placed in a stereotaxic frame (David Kopf Instruments) with the incisor bar set at −3.2 mm. Guide cannulas (PlasticsOne) were implanted according to the following coordinates, measured relative to the skull at bregma (Paxinos and Watson, 1998): anteroposterior −3.9 mm, lateral ± 1.9 mm, dorsoventral −3.0 mm. The cannulas were secured to the skull using dental acrylic and three jeweler screws. Obturators, cut to sit flush with the tip of the guide cannulas and with an outer diameter of 0.36 mm, were inserted into the guides and remained there except during infusions. A screw-on dust cap kept the obturators in place. At the completion of each surgery, antibiotic powder (Acramide; Dales Pharmaceuticals) was applied. Animals were given at least 7 days to recover prior to drug testing.

Infusion Procedure

Depending on the experiment, rats received bilateral infusions of either anti-BDNF (1 μg μl^{-1}/0.5 μl side; Millipore), sheep immunoglobulin G (IgG; 1 μg μl^{-1}/0.5 μl side; Millipore), oligonucleotides (ODNs; 4 nmol μl^{-1}/0.5 μl side; Sigma), human recombinant BDNF (0.5 μg μl^{-1}/0.5 μl side; Byoscience), or saline at different times during the SLR task. ODNs (Sigma) were high-performance liquid chromatography-purified phosphorothioate end-capped 18-mer sequences, resuspended in sterile saline to a concentration of 4 nmol μl^{-1}. Both ODNs were phosphorothioated on the three terminal bases of both 5′ and 3′ ends. This modification results in increased stability and less toxicity of the ODN (BDNF ASO, 5′-TCTTCCCCTTTTAATGGT-3′; BDNF MSO, 5′-ATACTTTCTGTTCTTGCC-3′). Both ODN sequences were subjected to a BLAST search on the National Center for Biotechnology Information BLAST server using the GenBank database. BDNF ASO is specific for rat BDNF mRNA. Control MSO sequence, which included the same 18 nucleotides as the ASO but in a scrambled order, did not generate any full matches to identified gene sequences in the database. Bilateral infusions were conducted simultaneously using two 5 μl Hamilton syringes that were connected to the infusion cannulas by propylene tubing. Syringes were driven by a Harvard Apparatus precision syringe pump, which delivered 0.5 μl to each hemisphere over 2 min. The infusion cannulas were left in place for an additional minute to allow for diffusion. At least 3 days were allowed for washout between repeated infusions.

Immunoblot Assays

After rats were sacrificed, brains were immediately frozen and the hippocampal DG, CA3, or CA1 regions were microdissected using a 1 mm section rat brain matrix (Braintree Scientific) and frozen on dry ice prior to storage at −80°C. Tissue was homogenized in ice-chilled buffer (20 mM Tris-HCL [pH 7.4], 0.32 M sucrose, 1 mM EDTA, 1 mM EGTA, 1 mM phenylmethanesulfonylfluoride, 10 mg/ml aprotinin, 15 mg/ml leupeptin, 10 mg/ml bacitracin, 10 mg/ml pepstatin, 15 mg/ml trypsin inhibitor, 50 mM NaF, and 1 mM sodium orthovanadate). Samples of homogenates (20 μg of protein) were subjected to 10% SDS-PAGE under reducing conditions. Proteins were transferred onto nitrocellulose membranes (Bio-Rad) in transfer buffer (25 mM Tris, 192 mM glycine, 20% v/v methanol) for 2 hr at 100 V. Western blots were performed by incubating membranes first with BDNF antibody (N20, 1:1,000; Santa Cruz Biotechnology), and then stripped and incubated with Zif268 (1:2,000, Santa Cruz Biotechnology) and actin antibodies (1:5,000, Santa Cruz Biotechnology). One nanogram of recombinant human BDNF was used as a standard for western blot (rhBDNF; Byoscience). Blots were developed using enhanced chemiluminescence (Thermo Fisher), visualized by the Chemidoc-It imaging system (UVP) and quantified using ImageJ software (National Institutes of Health). For analysis, optical density (OD) values and the band areas were obtained for each microdissected hippocampal sample for both the target protein (BDNF, Zif268) and the actin loading control. Each target OD value was normalized to its corresponding actin OD value and normalized levels were averaged for each condition. Data were analyzed using a one-way ANOVA followed by Newman-Keuls post hoc comparisons.

Apparatus

The circular open field (90 cm diameter × 45 cm high) was made of black plastic. It was situated in the middle of a dimly lit room and surrounded by three proximal spatial cues and distal standard furniture. The open field floor was covered with wood shavings. A video camera was positioned over the arena and sample and choice phases were recorded on to DVD for later analysis. The objects used were either soda cans or beer bottles from which the label had been removed. They were fixed to the floor of the open field with Blu-tack and cleaned with a 50% ethanol solution between sample and choice trials. Positions varied according to the experiment, with objects always placed along a circumference 15 cm away from the wall and 30 cm away from the center of the arena.

Cell PRESS Open ACCESS

Behavioral Procedures

Each rat was handled for 3 days and then habituated to the arena for 10 min a day for 5 days before exposure to the objects. For the SLR task, after habituation, rats were exposed to three identical objects (A1, A2, and A3) during a sample phase that lasted for 10 min. For the s-SLR, objects A2 and A3 were placed 50° apart (20.5 cm between them) and object A3 at an equal distance from the other two. For the d-SLR, objects A1, A2, and A3 were equidistant, 120° (49 cm between them) apart from each other. For the xs-SLR, A1 and A2 were separated by a 40° angle (15.4 cm between them). Twenty-four hours after the sample phase, rats were exposed to two new identical copies of the objects, named A4 and A5, for 5 min. New identical copies were used to prevent the use of olfactory cues. During this choice phase, object A4 was placed in a familiar location (same position as in the sample phase) and object A5 was placed in a novel location. For the s-SLR task, the novel location was defined as a position exactly in between the ones in which objects A2 and A3 were located during the sample phase (see schemes in Figures 1, 2, 3, 4, and 6). For the d-SLR task, object A4 was placed in a familiar location and object A5 in a position equidistant to the previous locations of A2 and A3 (see schemes in Figures 2, 3, and 4). One of the objects was always placed in a novel location, except during the choice phase for the "familiar" version of the SLR task (see Figure 1), in which the two objects (A4 and A5) were both placed in familiar locations. Results were expressed as a discrimination ratio that was calculated as the time exploring the object in the novel location minus the time exploring the object in the familiar location over total exploration time $[(t_{novel} - t_{familiar})/t_{total}]$. Absolute exploration times are shown in Tables S1 and S2. For the experiment shown in Figure 1, half of the rats were tested first in the "novel condition" and then in the "familiar condition," and the other half were tested first for the familiar and then for the novel conditions. Discrimination ratios were compared within subject using a paired t test. For experiments shown in Figures 2, 3, 4, and 6, rats were tested twice. In the first trial, half of the animals received drug injection and the other half received vehicle injection. In the second trial, they were injected with either drug or vehicle depending on what they had received in the first trial. For the behavioral experiments depicted in Figures 2D, 2E, 2F, 3, 4, and 6, discrimination ratios were compared within subject using a paired t test. For the experiments shown in Figure 2C, discrimination ratios were analyzed using a two-way repeated-measures ANOVA followed by Bonferroni post hoc comparisons. In all experiments, drug and vehicle injections were counterbalanced. See Supplemental Information for additional analysis.

For the experiment depicted in Figure 5, two identical objects (A1 and A2) were placed in the open field either 50° apart (small separation) or 120° apart (large separation). Different groups of rats were exposed to the small-separation condition, the large-separation condition, or the empty arena for 5 min. One hour after the exposure, rats were sacrificed by carbon dioxide inhalation to perform the protein extraction. For all experiments, exploration of a particular object was defined as the rat having its nose directed at the object at a distance of 2 cm or less, or touching the object with its nose. Rearing with the head oriented upward did not count as exploration. Climbing over or sitting on the objects was not included.

Histology

At the completion of behavioral testing, all rats except the ones used for experiments depicted in Figures 1 and 6 were anaesthetized by i.p. injection with 2 ml of Euthatal (Rhône Merieux) and perfused transcardially with PBS, followed by 10% neutral buffered formalin. The brains were removed and postfixed in formalin for at least 24 hr before being immersed in 20% sucrose solution until they sank. Sections 60 μm thick were cut on a freezing microtome encompassing the extent of the injector track. Every fifth section was mounted on a gelatin-coated glass slide and stained with cresyl violet. Slides were examined under a light microscope to verify the location of the injections.

Data Collection

Exploration was recorded by the experimenter using a computer program written in Visual Basic 6.0 (Microsoft). Two keys corresponded to the novel and familiar objects. Object exploration in both the sample and choice phases was recorded by pressing the appropriate key at the onset of a bout of exploration and then pressing it again at the offset.

SUPPLEMENTAL INFORMATION

Supplemental Information includes Supplemental Analysis and two tables and can be found with this article online at http://dx.doi.org/10.1016/j.celrep.2013.09.027.

ACKNOWLEDGMENTS

The authors would like to thank Dr. Henriette Van Praag, Dr. Lucas Pozzo-Miller, and Dr. Jess Nithianantarajah for helpful discussions about the manuscript and David Theobald for help with some experiments. We thank Justine Bonet and Andrika Morant for assistance with rat colony management. This work was funded by the Biotechnology and Biological Sciences Research Council (grant BB/G019002/1 to T.J.B., L.M.S., and P.B.). The research leading to these results has received support from the Innovative Medicine Initiative Joint Undertaking under grant agreement number 115008, of which resources are composed of EFPIA in-kind contribution and financial contribution from the European Union's Seventh Framework Programme (FP7/2007-2013). This work is also supported by the James S. McDonnell Foundation, Mather's Foundation, NIMH, Ellison Foundation, NINDS, NIMH, NIA, and JPB Foundation (to F.H.G.).

Received: April 6, 2013
Revised: July 24, 2013
Accepted: September 19, 2013
Published: October 24, 2013

REFERENCES

Aimone, J.B., Wiles, J., and Gage, F.H. (2009). Computational influence of adult neurogenesis on memory encoding. Neuron *61*, 187–202.

Alonso, M., Vianna, M.R., Depino, A.M., Mello e Souza, T., Pereira, P., Szapiro, G., Viola, H., Pitossi, F., Izquierdo, I., and Medina, J.H. (2002). BDNF-triggered events in the rat hippocampus are required for both short- and long-term memory formation. Hippocampus *12*, 551–560.

Bamji, S.X., Rico, B., Kimes, N., and Reichardt, L.F. (2006). BDNF mobilizes synaptic vesicles and enhances synapse formation by disrupting cadherin-beta-catenin interactions. J. Cell Biol. *174*, 289–299.

Bekinschtein, P., Cammarota, M., Igaz, L.M., Bevilaqua, L.R., Izquierdo, I., and Medina, J.H. (2007). Persistence of long-term memory storage requires a late protein synthesis- and BDNF- dependent phase in the hippocampus. Neuron *53*, 261–277.

Bekinschtein, P., Oomen, C.A., Saksida, L.M., and Bussey, T.J. (2011). Effects of environmental enrichment and voluntary exercise on neurogenesis, learning and memory, and pattern separation: BDNF as a critical variable? Semin. Cell Dev. Biol. *22*, 536–542.

Bergami, M., Rimondini, R., Santi, S., Blum, R., Götz, M., and Canossa, M. (2008). Deletion of TrkB in adult progenitors alters newborn neuron integration into hippocampal circuits and increases anxiety-like behavior. Proc. Natl. Acad. Sci. USA *105*, 15570–15575.

Burgess, N. (2008). Spatial cognition and the brain. Ann. N Y Acad. Sci. *1124*, 77–97.

Clelland, C.D., Choi, M., Romberg, C., Clemenson, G.D., Jr., Fragniere, A., Tyers, P., Jessberger, S., Saksida, L.M., Barker, R.A., Gage, F.H., and Bussey, T.J. (2009). A functional role for adult hippocampal neurogenesis in spatial pattern separation. Science *325*, 210–213.

Cunha, C., Brambilla, R., and Thomas, K.L. (2010). A simple role for BDNF in learning and memory? Front. Mol. Neurosci. *3*, 1.

Ennaceur, A., Neave, N., and Aggleton, J.P. (1997). Spontaneous object recognition and object location memory in rats: the effects of lesions in the cingulate cortices, the medial prefrontal cortex, the cingulum bundle and the fornix. Exp. Brain Res. *113*, 509–519.

Ge, S., Yang, C.H., Hsu, K.S., Ming, G.L., and Song, H. (2007). A critical period for enhanced synaptic plasticity in newly generated neurons of the adult brain. Neuron *54*, 559–566.

Gilbert, P.E., Kesner, R.P., and DeCoteau, W.E. (1998). Memory for spatial location: role of the hippocampus in mediating spatial pattern separation. J. Neurosci. *18*, 804–810.

Kang, H., Welcher, A.A., Shelton, D., and Schuman, E.M. (1997). Neurotrophins and time: different roles for TrkB signaling in hippocampal long-term potentiation. Neuron *19*, 653–664.

Karlsson, M.P., and Frank, L.M. (2009). Awake replay of remote experiences in the hippocampus. Nat. Neurosci. *12*, 913–918.

Korte, M., Carroll, P., Wolf, E., Brem, G., Thoenen, H., and Bonhoeffer, T. (1995). Hippocampal long-term potentiation is impaired in mice lacking brain-derived neurotrophic factor. Proc. Natl. Acad. Sci. USA *92*, 8856–8860.

Lee, J.L., Everitt, B.J., and Thomas, K.L. (2004). Independent cellular processes for hippocampal memory consolidation and reconsolidation. Science *304*, 839–843.

Leutgeb, J.K., Leutgeb, S., Moser, M.B., and Moser, E.I. (2007). Pattern separation in the dentate gyrus and CA3 of the hippocampus. Science *315*, 961–966.

Linnarsson, S., Björklund, A., and Ernfors, P. (1997). Learning deficit in BDNF mutant mice. Eur. J. Neurosci. *9*, 2581–2587.

McGaugh, J.L. (2000). Memory—a century of consolidation. Science *287*, 248–251.

McHugh, T.J., Jones, M.W., Quinn, J.J., Balthasar, N., Coppari, R., Elmquist, J.K., Lowell, B.B., Fanselow, M.S., Wilson, M.A., and Tonegawa, S. (2007). Dentate gyrus NMDA receptors mediate rapid pattern separation in the hippocampal network. Science *317*, 94–99.

Mizuno, M., Yamada, K., Olariu, A., Nawa, H., and Nabeshima, T. (2000). Involvement of brain-derived neurotrophic factor in spatial memory formation and maintenance in a radial arm maze test in rats. J. Neurosci. *20*, 7116–7121.

Nakashiba, T., Cushman, J.D., Pelkey, K.A., Renaudineau, S., Buhl, D.L., McHugh, T.J., Rodriguez Barrera, V., Chittajallu, R., Iwamoto, K.S., McBain, C.J., et al. (2012). Young dentate granule cells mediate pattern separation, whereas old granule cells facilitate pattern completion. Cell *149*, 188–201.

O'Reilly, R.C., and McClelland, J.L. (1994). Hippocampal conjunctive encoding, storage, and recall: avoiding a trade-off. Hippocampus *4*, 661–682.

Pang, P.T., Teng, H.K., Zaitsev, E., Woo, N.T., Sakata, K., Zhen, S., Teng, K.K., Yung, W.H., Hempstead, B.L., and Lu, B. (2004). Cleavage of proBDNF by tPA/plasmin is essential for long-term hippocampal plasticity. Science *306*, 487–491.

Paxinos, G., and Watson, C. (1998). The Rat Brain in Stereotaxic Coordinates, Fourth Edition (San Diego: Academic Press).

Peters, J., Dieppa-Perea, L.M., Melendez, L.M., and Quirk, G.J. (2010). Induction of fear extinction with hippocampal-infralimbic BDNF. Science *328*, 1288–1290.

Rolls, E.T., and Kesner, R.P. (2006). A computational theory of hippocampal function, and empirical tests of the theory. Prog. Neurobiol. *79*, 1–48.

Rossi, C., Angelucci, A., Costantin, L., Braschi, C., Mazzantini, M., Babbini, F., Fabbri, M.E., Tessarollo, L., Maffei, L., Berardi, N., and Caleo, M. (2006). Brain-derived neurotrophic factor (BDNF) is required for the enhancement of hippocampal neurogenesis following environmental enrichment. Eur. J. Neurosci. *24*, 1850–1856.

Scharfman, H., Goodman, J., Macleod, A., Phani, S., Antonelli, C., and Croll, S. (2005). Increased neurogenesis and the ectopic granule cells after intrahippocampal BDNF infusion in adult rats. Exp. Neurol. *192*, 348–356.

Schmidt-Hieber, C., Jonas, P., and Bischofberger, J. (2004). Enhanced synaptic plasticity in newly generated granule cells of the adult hippocampus. Nature *429*, 184–187.

Tyler, W.J., and Pozzo-Miller, L.D. (2001). BDNF enhances quantal neurotransmitter release and increases the number of docked vesicles at the active zones of hippocampal excitatory synapses. J. Neurosci. *21*, 4249–4258.

Warburton, E.C., Baird, A.L., Morgan, A., Muir, J.L., and Aggleton, J.P. (2000). Disconnecting hippocampal projections to the anterior thalamus produces deficits on tests of spatial memory in rats. Eur. J. Neurosci. *12*, 1714–1726.

Table S1. Total Exploration Times during the Sample Session for All Experiments

Fig n°	A1	A2	A3	A1	A2	A3
1C	36.2±3.5	38.5±1.7	44.2±3.7			
	IgG			anti-BDNF		
2C	28.3±4.0	33.0±5.0	33.9±6.8	27.7±5.1	33.7±3.9	27.7±5.1
	MSO			ASO		
3C	32.1±1.7	30.3±2.1	31.6±1.8	26.6±2.6	29.7±3.1	28.4±2.8
6B	26.7±2.2	26.8±2.6	25.7±2.4			

Results are expressed as mean ± SEM in seconds. A1, A2 and A3 represent the different locations as indicated in each one of the figures.

Table S2. Total Exploration Times during the Choice Session of the SLR Task for All Experiments

Results are expressed as mean ± SEM in seconds.

Fig n°	Novel location	Familiar location	Novel location	Familiar location	Novel location	Familiar location	Novel location	Familiar location
	Novel condition		Familiar condition					
1D	29.5±5.5	17.7±3.7	21.7±2.2	22.03±2.7				
	Small separation		Large separation					
1E	31.8±3.1	20.1±2.4	31.5±4.5	17.3±1.8				
	s-SLR				d-SLR			
	IgG		anti-BDNF		IgG		anti-BDNF	
2D	38.5±3.1	27.7±2.2	28.8±1.6	35.8±3.5	34.2±4.7	21.0±2.7	36.4±2.0	21.6±0.67
	s-SLR				d-SLR			
	IgG		anti-BDNF		IgG		anti-BDNF	
2E	26.1±2.2	17.4±1.5	23.0±2.3	25.5±3.5	37.4±4.6	21.3±2.8	37.4±2.4	22.7±1.3
2F	s-SLR							
	IgG		anti-BDNF					
	19.9±2.4	12.3±2.5	19.8±2.4	12.3±1.3				
	s-SLR				d-SLR			
	MSO		ASO		MSO		ASO	
3D	21.6±2.3	12.33±1.3	15.4±2.5	21.5±5.3	30.4±17.2	17.2±	31.2±5.7	18.2±3.3
	s-SLR							
	IgG		anti-BDNF					
4B	25.5±3.3	17.5±2.9	30.1±5.7	16.4±3.1				
	xs-SLR							
	Saline		BDNF					
6C	19.7±3.5	22.3±5.3	25.2±2.0	16.5±1.5				

Supplemental Analysis

In some experiments, namely those depicted in Figures 2D and 3D, infusion of the anti-BDNF antibody or the BDNF ASO in the s-SLR condition or saline in the xs-SLR condition produced numerically negative discrimination ratios. We thus analyzed whether these negative ratios were statistically significant. Both anti-BDNF and BDNF ASO values were not statistically different from zero ($p>0.05$ anti-BDNF vs 0 and $p>0.05$ BDNF ASO vs 0, One sample *t* test. In addition, we found that for the experiments shown in Figures 2D and 3D, control values were always significantly above zero ($p<0.01$ anti-IgG vs 0 and $p<0.001$ BDNF MSO vs 0, one sample *t* test). This analysis provides further evidence that the observed effects were due to the ability of the drugs to bring performance to chance levels, and were not driven by the apparent negative discrimination ratios observed in some instances.
